# Diytterbium(II) lithium indium(III) digermanide, Yb_2_LiInGe_2_

**DOI:** 10.1107/S1600536810014595

**Published:** 2010-04-28

**Authors:** Tae-Soo You, Svilen Bobev

**Affiliations:** a Department of Chemistry and Biochemistry, University of Delaware, Newark, DE 19716, USA

## Abstract

The title compound, Yb_2_LiInGe_2_, a new ordered quaternary inter­metallic phase, crystallizes with the ortho­rhom­bic Ca_2_LiInGe_2_ type (Pearson code *oP*24). The crystal structure contains six crystallographically unique sites in the asymmetric unit, all in special positions with site symmetry .*m*.. The structure is complex and based on [InGe_4_] tetra­hedra, which share corners in two directions, forming layers parallel to (001). Yb atoms fill square-pyramidal (Yb1) and octa­hedral (Yb2) inter­stices between the [InGe_4/2_] layers, while the small Li^+^ atoms fill tetra­hedral sites.

## Related literature

Isotypic *Ae*_2_LiInGe_2_ (*Ae* = Ca, Sr) compounds have been reported by Mao *et al.* (2001 ▶). Other related structures include Ca_2_CdSb_2_ and Yb_2_CdSb_2_ (Xia & Bobev, 2007 ▶), SrInGe and EuInGe (Mao *et al.*, 2002 ▶), (Eu_1-*x*_Ca_*x*_)_3_In_2_Ge_3_ and (Eu_1-*x*_Ca_*x*_)_4_In_3_Ge_4_ (You *et al.*, 2010 ▶), and (Sr_1-*x*_Ca_*x*_)_5_In_3_Ge_6_ (You & Bobev, 2010 ▶). *STRUCTURE TIDY* (Gelato & Parthé, 1987 ▶) was used for standardization of the atomic coordinates.

## Experimental

### 

#### Crystal data

Yb_2_LiInGe_2_
*M* *_r_* = 613.02
Orthorhombic, 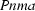

*a* = 7.182 (3) Å
*b* = 4.3899 (18) Å
*c* = 16.758 (7) Å
*V* = 528.3 (4) Å^3^
*Z* = 4
Mo *K*α radiation
μ = 50.42 mm^−1^
*T* = 200 K
0.04 × 0.02 × 0.02 mm

#### Data collection

Bruker SMART APEX diffractometer
Absorption correction: multi-scan (*SADABS*; Bruker, 2002 ▶) *T*_min_ = 0.258, *T*_max_ = 0.365
6805 measured reflections
735 independent reflections
623 reflections with *I* > 2σ(*I*)
*R*_int_ = 0.090

#### Refinement

*R*[*F* ^2^ > 2σ(*F* ^2^)] = 0.029
*wR*(*F* ^2^) = 0.059
*S* = 1.11
735 reflections
35 parameters
Δρ_max_ = 2.10 e Å^−3^
Δρ_min_ = −2.86 e Å^−3^

### 

Data collection: *SMART* (Bruker, 2002 ▶); cell refinement: *SAINT* (Bruker, 2002 ▶); data reduction: *SAINT*; program(s) used to solve structure: *SHELXTL* (Sheldrick, 2008 ▶); program(s) used to refine structure: *SHELXTL*; molecular graphics: *DIAMOND* (Brandenburg, 1999 ▶); software used to prepare material for publication: *SHELXTL*.

## Supplementary Material

Crystal structure: contains datablocks global, I. DOI: 10.1107/S1600536810014595/wm2327sup1.cif

Structure factors: contains datablocks I. DOI: 10.1107/S1600536810014595/wm2327Isup2.hkl

Additional supplementary materials:  crystallographic information; 3D view; checkCIF report

## Figures and Tables

**Table 1:** Selected bond lengths (Å)

In—Ge1^i^	2.7803 (13)
In—Ge1^ii^	2.7803 (13)
In—Ge2^iii^	2.809 (2)
In—Ge2	2.8203 (19)

Symmetry codes: (i) 
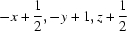
; (ii) 
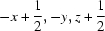
; (iii) 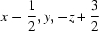.

**Fig. 1.**
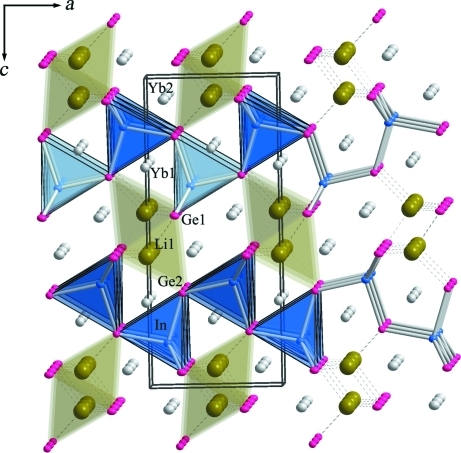
Combined ellipsoid and polyhedral representations of the crystal structure of orthorhombic Yb2LiInGe2, viewed along [010]. Color code: Yb - light grey, Li - dark yellow, In - light blue, and Ge - magenta. Ellipsoids are drawn at the 90% probability level.

## supplementary crystallographic information

### Comment

During our exploratory investigations of lithium-containing germanides using molten indium as a metal flux, the quaternary compound Yb_2_LiInGe_2_ was obtained for the first time.  It crystallizes in space group *Pnma* and is isostructural with *Ae*_2_LiInGe_2_ (*Ae* = Ca and Sr) compounds which were reported previously by Mao *et al.* (2001). This finding implies that this series can probably be extended towards other lanthanide metals, which likewise exhibit a stable oxidation state of +II, such as Eu for example.

The crystal structure of the title compound can be readily described as consisting of puckered polyanionic layers of corner-shared [InGe_4_] tetrahedra, running parallel to the *ab* plane and alternately stacked along the *c* axis (Figure 1). Yb and Li atoms, in turn, can be viewed simply as "electron donors", which provide the electrons to fill the valence shells of In and Ge, as well as "spacers" that separate the [InGe_4/2_]^5-^ polyanionic layers.

The In—Ge bond distances observed within the [InGe_4_] tetrahedron range from 2.7803 (13) to 2.8203 (13) Å, and are comparable to those in other indium germanides such as Ca_2_LiInGe_2_ (2.806 (1) - 2.838 (1)Å; Mao *et al.*, 2001), Sr_2_LiInGe_2_ (2.885 (1) - 2.926 (1) Å; Mao *et al.*, 2001), EuInGe (2.751 (1) Å; Mao *et al.*, 2002), SrInGe (2.780 Å; Mao *et al.*, 2002), as well as the recently reported (Eu_1-_*_x_*Ca*_x_*)_3_In_2_Ge_3_ (2.760 (2) - 2.869 (1) Å), (Eu_1-_*_x_*Ca*_x_*)_4_In_3_Ge_4_ (2.755 (2) - 2.887 (1) Å; You *et al.*, 2010), and (Sr_1-_*_x_*Ca*_x_*)_5_In_3_Ge_6_ (2.672 (2) - 2.877 (3) Å; You & Bobev, 2010). In the absence of direct In—In or Ge—Ge bonding, the formula of the title compound can be rationalized as follows: [(Yb^2+^)_2_(Li^+^)][(4*b*-In^-^) (2*b*-Ge^2-^)_2_]. Here, the In atom is tetrahedrally surrounded by four Ge atoms (Ge1 × 2 and Ge2 × 2) and is therefore assigned a formal charge of "-1", while the Ge atoms are 2-bonded, carrying a formal charge of "-2" each (4-bonded and 2-bonded atoms are denoted as 4*b*- and 2*b*-, respectively).

Interestingly, the structure of the title compound closely resembles the structure of one of our previously reported antimonides, *viz*. Ca_2_CdSb_2_ (Xia & Bobev, 2007). The latter structure is also made up of corrugated layers of corner-shared [CdSb_4_] tetrahedra, with Ca^2+^ cations filling the space between them. The obvious difference between these two structure types is the addition of Li atoms in Yb_2_LiInGe_2_, filling small tetrahedral holes between the layers (Figure 1).

### Experimental

The flux reaction was carried out in a 2 cm^3^ alumina crucible, using a total *ca* 500 mg mixture of the elements (Yb and Ge from Alfa, Li from Sigma-Aldrich), which was then topped off with *ca* 2 grams of In (Alfa, shots) acting as a metal flux. The crucible was subsequently enclosed and flame-sealed in an evacuated fused silica ampoule, and then was heated at 120 Kh^-1^ to 1223 K, kept there for 10 h, cooled to 573 K, where the excess In was removed by centrifugation.

### Refinement

Displacement parameters for all atoms were refined anisotropically except those of Li. The maximum residual electron density lies 0.87 Å from Yb1, and the minimum residual electron density lies 1.97 Å from Ge2.  The atomic coordinates have been standardized with the aid of STRUCTURE TIDY (Gelato & Parthé, 1987).

### Crystal data

**Table:**

Yb_2_LiInGe_2_	*F*(000) = 1024
*M**_r_* = 613.02	*D*_x_ = 7.707 Mg m^−^^3^
Orthorhombic, *P**n**m**a*	Mo *K*α radiation, λ = 0.71073 Å
Hall symbol: -P 2ac 2n	Cell parameters from 735 reflections
*a* = 7.182 (3) Å	θ = 2.4–28.2°
*b* = 4.3899 (18) Å	µ = 50.42 mm^−^^1^
*c* = 16.758 (7) Å	*T* = 200 K
*V* = 528.3 (4) Å^3^	Needle, grey-silver
*Z* = 4	0.04 × 0.02 × 0.02 mm

### Data collection

**Table:**

Bruker SMART APEX diffractometer	735 independent reflections
Radiation source: fine-focus sealed tube	623 reflections with *I* > 2σ(*I*)
graphite	*R*_int_ = 0.090
ω scans	θ_max_ = 28.2°, θ_min_ = 2.4°
Absorption correction: multi-scan (*SADABS*; Bruker, 2002)	*h* = −9→9
*T*_min_ = 0.258, *T*_max_ = 0.365	*k* = −5→5
6805 measured reflections	*l* = −22→22

### Refinement

**Table:**

Refinement on *F*^2^	Primary atom site location: structure-invariant direct methods
Least-squares matrix: full	Secondary atom site location: difference Fourier map
*R*[*F*^2^ > 2σ(*F*^2^)] = 0.029	*w* = 1/[σ^2^(*F*_o_^2^) + (0.*P*)^2^ + 1.4209*P*] where *P* = (*F*_o_^2^ + 2*F*_c_^2^)/3
*wR*(*F*^2^) = 0.059	(Δ/σ)_max_ < 0.001
*S* = 1.11	Δρ_max_ = 2.10 e Å^−^^3^
735 reflections	Δρ_min_ = −2.86 e Å^−^^3^
35 parameters	Extinction correction: *SHELXTL* (Sheldrick, 2008), Fc^*^=kFc[1+0.001xFc^2^λ^3^/sin(2θ)]^-1/4^
0 restraints	Extinction coefficient: 0.00117 (14)

### Special details

**Table:**

Experimental. Selected in the glove box, crystals were put in a Paratone N oil and cut to the desired dimensions. The chosen crystal was mounted on a tip of a glass fiber and quickly transferred onto the goniometer. The crystal was kept under a cold nitrogen stream to protect from the ambient air and moisture.Data collection is performed with four batch runs at φ = 0.00 ° (607 frames), at φ = 90.00 ° (607 frames), at φ = 180.00 ° (607 frames), and at φ = 270.00 (607 frames). Frame width = 0.30 ° in ω. Data are merged and treated with multi-scan absorption corrections.
Geometry. All esds (except the esd in the dihedral angle between two l.s. planes) are estimated using the full covariance matrix. The cell esds are taken into account individually in the estimation of esds in distances, angles and torsion angles; correlations between esds in cell parameters are only used when they are defined by crystal symmetry. An approximate (isotropic) treatment of cell esds is used for estimating esds involving l.s. planes.
Refinement. Refinement of *F*^2^ against ALL reflections. The weighted *R*-factor wR and goodness of fit *S* are based on *F*^2^, conventional *R*-factors *R* are based on *F*, with *F* set to zero for negative *F*^2^. The threshold expression of *F*^2^ > σ(*F*^2^) is used only for calculating *R*-factors(gt) etc. and is not relevant to the choice of reflections for refinement. *R*-factors based on *F*^2^ are statistically about twice as large as those based on *F*, and *R*- factors based on ALL data will be even larger.

### Fractional atomic coordinates and isotropic or equivalent isotropic displacement parameters (Å^2^)

**Table:**

	*x*	*y*	*z*	*U*_iso_*/*U*_eq_	
Yb1	0.01067 (8)	0.2500	0.27892 (3)	0.00935 (17)	
Yb2	0.15804 (9)	0.2500	0.06161 (3)	0.01085 (17)	
In	0.15783 (13)	0.2500	0.84697 (5)	0.0079 (2)	
Ge1	0.22805 (19)	0.2500	0.43629 (8)	0.0078 (3)	
Ge2	0.27452 (18)	0.2500	0.68627 (8)	0.0065 (3)	
Li1	0.011 (4)	0.2500	0.5672 (16)	0.023 (6)*	

### Atomic displacement parameters (Å^2^)

**Table:**

	*U*^11^	*U*^22^	*U*^33^	*U*^12^	*U*^13^	*U*^23^
Yb1	0.0083 (3)	0.0110 (3)	0.0088 (3)	0.000	0.0001 (2)	0.000
Yb2	0.0116 (3)	0.0121 (3)	0.0089 (3)	0.000	−0.0001 (2)	0.000
In	0.0076 (5)	0.0102 (4)	0.0060 (5)	0.000	−0.0004 (3)	0.000
Ge1	0.0094 (7)	0.0087 (6)	0.0052 (7)	0.000	0.0004 (5)	0.000
Ge2	0.0058 (7)	0.0083 (6)	0.0054 (7)	0.000	0.0002 (5)	0.000

### Geometric parameters (Å, °)

**Table:**

Yb1—Ge2^i^	3.0583 (13)	In—Yb1^viii^	3.4331 (12)
Yb1—Ge2^ii^	3.0583 (13)	In—Yb1^ix^	3.4331 (12)
Yb1—Ge1	3.0646 (18)	In—Yb2^i^	3.5087 (12)
Yb1—Ge2^iii^	3.0997 (13)	In—Yb2^ii^	3.5087 (12)
Yb1—Ge2^iv^	3.0997 (13)	In—Yb2^xii^	3.5969 (18)
Yb1—In^i^	3.2760 (12)	Ge1—Li1	2.69 (3)
Yb1—In^ii^	3.2760 (12)	Ge1—In^iv^	2.7803 (13)
Yb1—Li1^ii^	3.39 (2)	Ge1—In^iii^	2.7803 (13)
Yb1—Li1^i^	3.39 (2)	Ge1—Li1^i^	2.786 (17)
Yb1—In^iii^	3.4331 (12)	Ge1—Li1^ii^	2.786 (17)
Yb1—In^iv^	3.4331 (12)	Ge1—Yb2^vi^	3.0883 (19)
Yb1—Yb2^v^	3.6817 (13)	Ge1—Yb2^viii^	3.1460 (13)
Yb2—Ge2^iii^	3.0686 (13)	Ge1—Yb2^ix^	3.1460 (13)
Yb2—Ge2^iv^	3.0686 (13)	Ge2—Li1	2.75 (3)
Yb2—Ge1^v^	3.088 (2)	Ge2—In^xi^	2.809 (2)
Yb2—Ge1^iv^	3.1460 (13)	Ge2—Yb1^i^	3.0583 (13)
Yb2—Ge1^iii^	3.1460 (13)	Ge2—Yb1^ii^	3.0583 (13)
Yb2—Li1^iii^	3.24 (2)	Ge2—Yb2^ix^	3.0686 (13)
Yb2—Li1^iv^	3.24 (2)	Ge2—Yb2^viii^	3.0686 (13)
Yb2—Li1^vi^	3.33 (3)	Ge2—Yb1^viii^	3.0997 (13)
Yb2—In^i^	3.5087 (12)	Ge2—Yb1^ix^	3.0997 (13)
Yb2—In^ii^	3.5087 (12)	Li1—Ge1^i^	2.786 (17)
Yb2—In^vii^	3.5969 (18)	Li1—Ge1^ii^	2.786 (17)
Yb2—Yb1^vi^	3.6817 (13)	Li1—In^x^	2.91 (3)
In—Ge1^viii^	2.7803 (13)	Li1—Li1^i^	3.15 (4)
In—Ge1^ix^	2.7803 (13)	Li1—Li1^ii^	3.15 (4)
In—Ge2^x^	2.809 (2)	Li1—Yb2^viii^	3.24 (2)
In—Ge2	2.8203 (19)	Li1—Yb2^ix^	3.24 (2)
In—Li1^xi^	2.91 (3)	Li1—Yb2^v^	3.33 (3)
In—Yb1^i^	3.2760 (12)	Li1—Yb1^ii^	3.39 (2)
In—Yb1^ii^	3.2760 (12)	Li1—Yb1^i^	3.39 (2)
			
Ge2^i^—Yb1—Ge2^ii^	91.73 (5)	Ge1^viii^—In—Yb2^i^	116.71 (5)
Ge2^i^—Yb1—Ge1	100.19 (4)	Ge1^ix^—In—Yb2^i^	57.43 (4)
Ge2^ii^—Yb1—Ge1	100.19 (4)	Ge2^x^—In—Yb2^i^	56.83 (3)
Ge2^i^—Yb1—Ge2^iii^	159.57 (3)	Ge2—In—Yb2^i^	127.52 (3)
Ge2^ii^—Yb1—Ge2^iii^	85.45 (3)	Li1^xi^—In—Yb2^i^	110.3 (4)
Ge1—Yb1—Ge2^iii^	100.22 (4)	Yb1^i^—In—Yb2^i^	67.86 (3)
Ge2^i^—Yb1—Ge2^iv^	85.45 (3)	Yb1^ii^—In—Yb2^i^	117.48 (4)
Ge2^ii^—Yb1—Ge2^iv^	159.57 (3)	Yb1^viii^—In—Yb2^i^	173.39 (3)
Ge1—Yb1—Ge2^iv^	100.22 (4)	Yb1^ix^—In—Yb2^i^	101.15 (3)
Ge2^iii^—Yb1—Ge2^iv^	90.16 (5)	Ge1^viii^—In—Yb2^ii^	57.43 (4)
Ge2^i^—Yb1—In^i^	52.74 (3)	Ge1^ix^—In—Yb2^ii^	116.71 (4)
Ge2^ii^—Yb1—In^i^	110.87 (4)	Ge2^x^—In—Yb2^ii^	56.83 (3)
Ge1—Yb1—In^i^	137.93 (2)	Ge2—In—Yb2^ii^	127.52 (3)
Ge2^iii^—Yb1—In^i^	109.62 (4)	Li1^xi^—In—Yb2^ii^	110.3 (4)
Ge2^iv^—Yb1—In^i^	52.18 (4)	Yb1^i^—In—Yb2^ii^	117.48 (4)
Ge2^i^—Yb1—In^ii^	110.87 (4)	Yb1^ii^—In—Yb2^ii^	67.86 (3)
Ge2^ii^—Yb1—In^ii^	52.74 (3)	Yb1^viii^—In—Yb2^ii^	101.15 (3)
Ge1—Yb1—In^ii^	137.93 (2)	Yb1^ix^—In—Yb2^ii^	173.39 (3)
Ge2^iii^—Yb1—In^ii^	52.18 (4)	Yb2^i^—In—Yb2^ii^	77.45 (4)
Ge2^iv^—Yb1—In^ii^	109.62 (4)	Ge1^viii^—In—Yb2^xii^	57.42 (3)
In^i^—Yb1—In^ii^	84.13 (4)	Ge1^ix^—In—Yb2^xii^	57.42 (3)
Ge2^i^—Yb1—Li1^ii^	106.8 (4)	Ge2^x^—In—Yb2^xii^	101.46 (4)
Ge2^ii^—Yb1—Li1^ii^	50.2 (4)	Ge2—In—Yb2^xii^	162.69 (4)
Ge1—Yb1—Li1^ii^	50.8 (4)	Li1^xi^—In—Yb2^xii^	60.4 (5)
Ge2^iii^—Yb1—Li1^ii^	86.9 (3)	Yb1^i^—In—Yb2^xii^	130.10 (2)
Ge2^iv^—Yb1—Li1^ii^	149.6 (4)	Yb1^ii^—In—Yb2^xii^	130.10 (2)
In^i^—Yb1—Li1^ii^	155.1 (4)	Yb1^viii^—In—Yb2^xii^	109.38 (3)
In^ii^—Yb1—Li1^ii^	92.3 (3)	Yb1^ix^—In—Yb2^xii^	109.38 (3)
Ge2^i^—Yb1—Li1^i^	50.2 (4)	Yb2^i^—In—Yb2^xii^	64.13 (2)
Ge2^ii^—Yb1—Li1^i^	106.8 (4)	Yb2^ii^—In—Yb2^xii^	64.13 (2)
Ge1—Yb1—Li1^i^	50.8 (4)	Li1—Ge1—In^iv^	127.56 (6)
Ge2^iii^—Yb1—Li1^i^	149.6 (4)	Li1—Ge1—In^iii^	127.56 (6)
Ge2^iv^—Yb1—Li1^i^	86.9 (3)	In^iv^—Ge1—In^iii^	104.27 (6)
In^i^—Yb1—Li1^i^	92.3 (3)	Li1—Ge1—Li1^i^	70.1 (7)
In^ii^—Yb1—Li1^i^	155.1 (4)	In^iv^—Ge1—Li1^i^	63.1 (5)
Li1^ii^—Yb1—Li1^i^	80.7 (6)	In^iii^—Ge1—Li1^i^	142.4 (5)
Ge2^i^—Yb1—In^iii^	149.31 (4)	Li1—Ge1—Li1^ii^	70.1 (7)
Ge2^ii^—Yb1—In^iii^	86.68 (4)	In^iv^—Ge1—Li1^ii^	142.4 (5)
Ge1—Yb1—In^iii^	50.28 (2)	In^iii^—Ge1—Li1^ii^	63.1 (5)
Ge2^iii^—Yb1—In^iii^	50.84 (3)	Li1^i^—Ge1—Li1^ii^	104.0 (9)
Ge2^iv^—Yb1—In^iii^	105.90 (4)	Li1—Ge1—Yb1	113.9 (6)
In^i^—Yb1—In^iii^	153.95 (3)	In^iv^—Ge1—Yb1	71.75 (4)
In^ii^—Yb1—In^iii^	92.39 (3)	In^iii^—Ge1—Yb1	71.75 (4)
Li1^ii^—Yb1—In^iii^	50.6 (4)	Li1^i^—Ge1—Yb1	70.6 (5)
Li1^i^—Yb1—In^iii^	101.1 (4)	Li1^ii^—Ge1—Yb1	70.6 (5)
Ge2^i^—Yb1—In^iv^	86.68 (4)	Li1—Ge1—Yb2^vi^	124.8 (6)
Ge2^ii^—Yb1—In^iv^	149.31 (4)	In^iv^—Ge1—Yb2^vi^	73.22 (3)
Ge1—Yb1—In^iv^	50.28 (2)	In^iii^—Ge1—Yb2^vi^	73.22 (3)
Ge2^iii^—Yb1—In^iv^	105.90 (4)	Li1^i^—Ge1—Yb2^vi^	128.0 (4)
Ge2^iv^—Yb1—In^iv^	50.84 (3)	Li1^ii^—Ge1—Yb2^vi^	128.0 (4)
In^i^—Yb1—In^iv^	92.39 (3)	Yb1—Ge1—Yb2^vi^	121.28 (5)
In^ii^—Yb1—In^iv^	153.95 (3)	Li1—Ge1—Yb2^viii^	66.8 (4)
Li1^ii^—Yb1—In^iv^	101.1 (4)	In^iv^—Ge1—Yb2^viii^	146.46 (6)
Li1^i^—Yb1—In^iv^	50.6 (4)	In^iii^—Ge1—Yb2^viii^	74.45 (4)
In^iii^—Yb1—In^iv^	79.49 (4)	Li1^i^—Ge1—Yb2^viii^	136.4 (5)
Ge2^i^—Yb1—Yb2^v^	53.19 (3)	Li1^ii^—Ge1—Yb2^viii^	67.9 (5)
Ge2^ii^—Yb1—Yb2^v^	53.19 (3)	Yb1—Ge1—Yb2^viii^	134.98 (3)
Ge1—Yb1—Yb2^v^	74.08 (4)	Yb2^vi^—Ge1—Yb2^viii^	74.48 (3)
Ge2^iii^—Yb1—Yb2^v^	134.90 (2)	Li1—Ge1—Yb2^ix^	66.8 (4)
Ge2^iv^—Yb1—Yb2^v^	134.90 (2)	In^iv^—Ge1—Yb2^ix^	74.45 (4)
In^i^—Yb1—Yb2^v^	102.32 (3)	In^iii^—Ge1—Yb2^ix^	146.46 (6)
In^ii^—Yb1—Yb2^v^	102.32 (3)	Li1^i^—Ge1—Yb2^ix^	67.9 (5)
Li1^ii^—Yb1—Yb2^v^	54.3 (4)	Li1^ii^—Ge1—Yb2^ix^	136.4 (5)
Li1^i^—Yb1—Yb2^v^	54.3 (4)	Yb1—Ge1—Yb2^ix^	134.98 (3)
In^iii^—Yb1—Yb2^v^	103.64 (3)	Yb2^vi^—Ge1—Yb2^ix^	74.48 (3)
In^iv^—Yb1—Yb2^v^	103.64 (3)	Yb2^viii^—Ge1—Yb2^ix^	88.48 (5)
Ge2^iii^—Yb2—Ge2^iv^	91.33 (5)	Li1—Ge2—In^xi^	122.1 (6)
Ge2^iii^—Yb2—Ge1^v^	98.63 (3)	Li1—Ge2—In	119.2 (6)
Ge2^iv^—Yb2—Ge1^v^	98.63 (3)	In^xi^—Ge2—In	118.72 (5)
Ge2^iii^—Yb2—Ge1^iv^	155.85 (4)	Li1—Ge2—Yb1^i^	71.2 (4)
Ge2^iv^—Yb2—Ge1^iv^	85.09 (4)	In^xi^—Ge2—Yb1^i^	133.97 (2)
Ge1^v^—Yb2—Ge1^iv^	105.52 (3)	In—Ge2—Yb1^i^	67.60 (3)
Ge2^iii^—Yb2—Ge1^iii^	85.09 (4)	Li1—Ge2—Yb1^ii^	71.2 (4)
Ge2^iv^—Yb2—Ge1^iii^	155.85 (4)	In^xi^—Ge2—Yb1^ii^	133.97 (2)
Ge1^v^—Yb2—Ge1^iii^	105.52 (3)	In—Ge2—Yb1^ii^	67.60 (3)
Ge1^iv^—Yb2—Ge1^iii^	88.48 (5)	Yb1^i^—Ge2—Yb1^ii^	91.73 (5)
Ge2^iii^—Yb2—Li1^iii^	51.6 (4)	Li1—Ge2—Yb2^ix^	67.4 (4)
Ge2^iv^—Yb2—Li1^iii^	110.4 (4)	In^xi^—Ge2—Yb2^ix^	73.16 (3)
Ge1^v^—Yb2—Li1^iii^	137.2 (3)	In—Ge2—Yb2^ix^	134.18 (3)
Ge1^iv^—Yb2—Li1^iii^	107.5 (4)	Yb1^i^—Ge2—Yb2^ix^	73.87 (3)
Ge1^iii^—Yb2—Li1^iii^	49.9 (4)	Yb1^ii^—Ge2—Yb2^ix^	138.50 (5)
Ge2^iii^—Yb2—Li1^iv^	110.4 (4)	Li1—Ge2—Yb2^viii^	67.4 (4)
Ge2^iv^—Yb2—Li1^iv^	51.6 (4)	In^xi^—Ge2—Yb2^viii^	73.16 (3)
Ge1^v^—Yb2—Li1^iv^	137.2 (3)	In—Ge2—Yb2^viii^	134.18 (3)
Ge1^iv^—Yb2—Li1^iv^	49.9 (4)	Yb1^i^—Ge2—Yb2^viii^	138.50 (5)
Ge1^iii^—Yb2—Li1^iv^	107.5 (4)	Yb1^ii^—Ge2—Yb2^viii^	73.87 (3)
Li1^iii^—Yb2—Li1^iv^	85.4 (7)	Yb2^ix^—Ge2—Yb2^viii^	91.33 (5)
Ge2^iii^—Yb2—Li1^vi^	108.7 (3)	Li1—Ge2—Yb1^viii^	134.90 (3)
Ge2^iv^—Yb2—Li1^vi^	108.7 (3)	In^xi^—Ge2—Yb1^viii^	67.14 (3)
Ge1^v^—Yb2—Li1^vi^	140.2 (5)	In—Ge2—Yb1^viii^	70.71 (3)
Ge1^iv^—Yb2—Li1^vi^	50.9 (2)	Yb1^i^—Ge2—Yb1^viii^	138.24 (5)
Ge1^iii^—Yb2—Li1^vi^	50.9 (2)	Yb1^ii^—Ge2—Yb1^viii^	74.31 (3)
Li1^iii^—Yb2—Li1^vi^	57.3 (6)	Yb2^ix^—Ge2—Yb1^viii^	140.26 (5)
Li1^iv^—Yb2—Li1^vi^	57.3 (6)	Yb2^viii^—Ge2—Yb1^viii^	75.87 (3)
Ge2^iii^—Yb2—In^i^	104.61 (4)	Li1—Ge2—Yb1^ix^	134.90 (3)
Ge2^iv^—Yb2—In^i^	50.01 (3)	In^xi^—Ge2—Yb1^ix^	67.14 (3)
Ge1^v^—Yb2—In^i^	49.35 (3)	In—Ge2—Yb1^ix^	70.71 (3)
Ge1^iv^—Yb2—In^i^	91.33 (3)	Yb1^i^—Ge2—Yb1^ix^	74.31 (3)
Ge1^iii^—Yb2—In^i^	153.64 (4)	Yb1^ii^—Ge2—Yb1^ix^	138.24 (5)
Li1^iii^—Yb2—In^i^	152.4 (5)	Yb2^ix^—Ge2—Yb1^ix^	75.87 (3)
Li1^iv^—Yb2—In^i^	92.2 (4)	Yb2^viii^—Ge2—Yb1^ix^	140.26 (5)
Li1^vi^—Yb2—In^i^	140.83 (7)	Yb1^viii^—Ge2—Yb1^ix^	90.16 (5)
Ge2^iii^—Yb2—In^ii^	50.01 (3)	Ge1—Li1—Ge2	101.1 (9)
Ge2^iv^—Yb2—In^ii^	104.61 (4)	Ge1—Li1—Ge1^i^	109.9 (7)
Ge1^v^—Yb2—In^ii^	49.35 (3)	Ge2—Li1—Ge1^i^	116.0 (6)
Ge1^iv^—Yb2—In^ii^	153.64 (4)	Ge1—Li1—Ge1^ii^	109.9 (7)
Ge1^iii^—Yb2—In^ii^	91.33 (3)	Ge2—Li1—Ge1^ii^	116.0 (6)
Li1^iii^—Yb2—In^ii^	92.2 (4)	Ge1^i^—Li1—Ge1^ii^	104.0 (9)
Li1^iv^—Yb2—In^ii^	152.4 (5)	Ge1—Li1—In^x^	155.0 (11)
Li1^vi^—Yb2—In^ii^	140.83 (7)	Ge2—Li1—In^x^	103.9 (8)
In^i^—Yb2—In^ii^	77.45 (4)	Ge1^i^—Li1—In^x^	58.3 (5)
Ge2^iii^—Yb2—In^vii^	132.91 (3)	Ge1^ii^—Li1—In^x^	58.3 (5)
Ge2^iv^—Yb2—In^vii^	132.91 (3)	Ge1—Li1—Li1^i^	56.3 (8)
Ge1^v^—Yb2—In^vii^	90.63 (3)	Ge2—Li1—Li1^i^	123.5 (10)
Ge1^iv^—Yb2—In^vii^	48.13 (3)	Ge1^i^—Li1—Li1^i^	53.5 (5)
Ge1^iii^—Yb2—In^vii^	48.13 (3)	Ge1^ii^—Li1—Li1^i^	120.3 (13)
Li1^iii^—Yb2—In^vii^	91.7 (5)	In^x^—Li1—Li1^i^	108.1 (11)
Li1^iv^—Yb2—In^vii^	91.7 (5)	Ge1—Li1—Li1^ii^	56.3 (8)
Li1^vi^—Yb2—In^vii^	49.6 (5)	Ge2—Li1—Li1^ii^	123.5 (10)
In^i^—Yb2—In^vii^	115.87 (2)	Ge1^i^—Li1—Li1^ii^	120.3 (13)
In^ii^—Yb2—In^vii^	115.87 (2)	Ge1^ii^—Li1—Li1^ii^	53.5 (5)
Ge2^iii^—Yb2—Yb1^vi^	52.94 (3)	In^x^—Li1—Li1^ii^	108.1 (11)
Ge2^iv^—Yb2—Yb1^vi^	52.94 (3)	Li1^i^—Li1—Li1^ii^	88.4 (13)
Ge1^v^—Yb2—Yb1^vi^	132.81 (4)	Ge1—Li1—Yb2^viii^	63.3 (5)
Ge1^iv^—Yb2—Yb1^vi^	107.80 (4)	Ge2—Li1—Yb2^viii^	61.0 (5)
Ge1^iii^—Yb2—Yb1^vi^	107.80 (4)	Ge1^i^—Li1—Yb2^viii^	170.3 (8)
Li1^iii^—Yb2—Yb1^vi^	58.2 (4)	Ge1^ii^—Li1—Yb2^viii^	85.27 (12)
Li1^iv^—Yb2—Yb1^vi^	58.2 (4)	In^x^—Li1—Yb2^viii^	130.8 (5)
Li1^vi^—Yb2—Yb1^vi^	87.0 (5)	Li1^i^—Li1—Yb2^viii^	119.2 (13)
In^i^—Yb2—Yb1^vi^	97.34 (3)	Li1^ii^—Li1—Yb2^viii^	62.8 (6)
In^ii^—Yb2—Yb1^vi^	97.34 (3)	Ge1—Li1—Yb2^ix^	63.3 (5)
In^vii^—Yb2—Yb1^vi^	136.56 (3)	Ge2—Li1—Yb2^ix^	61.0 (5)
Ge1^viii^—In—Ge1^ix^	104.27 (6)	Ge1^i^—Li1—Yb2^ix^	85.27 (12)
Ge1^viii^—In—Ge2^x^	113.31 (4)	Ge1^ii^—Li1—Yb2^ix^	170.3 (8)
Ge1^ix^—In—Ge2^x^	113.31 (4)	In^x^—Li1—Yb2^ix^	130.8 (5)
Ge1^viii^—In—Ge2	115.24 (4)	Li1^i^—Li1—Yb2^ix^	62.8 (6)
Ge1^ix^—In—Ge2	115.24 (4)	Li1^ii^—Li1—Yb2^ix^	119.2 (13)
Ge2^x^—In—Ge2	95.85 (4)	Yb2^viii^—Li1—Yb2^ix^	85.4 (6)
Ge1^viii^—In—Li1^xi^	58.5 (2)	Ge1—Li1—Yb2^v^	85.0 (7)
Ge1^ix^—In—Li1^xi^	58.5 (2)	Ge2—Li1—Yb2^v^	173.9 (10)
Ge2^x^—In—Li1^xi^	161.9 (5)	Ge1^i^—Li1—Yb2^v^	61.2 (5)
Ge2—In—Li1^xi^	102.3 (5)	Ge1^ii^—Li1—Yb2^v^	61.2 (5)
Ge1^viii^—In—Yb1^i^	169.90 (3)	In^x^—Li1—Yb2^v^	70.0 (6)
Ge1^ix^—In—Yb1^i^	85.79 (4)	Li1^i^—Li1—Yb2^v^	59.9 (8)
Ge2^x^—In—Yb1^i^	60.68 (3)	Li1^ii^—Li1—Yb2^v^	59.9 (9)
Ge2—In—Yb1^i^	59.66 (3)	Yb2^viii^—Li1—Yb2^v^	122.7 (6)
Li1^xi^—In—Yb1^i^	129.7 (3)	Yb2^ix^—Li1—Yb2^v^	122.7 (6)
Ge1^viii^—In—Yb1^ii^	85.79 (4)	Ge1—Li1—Yb1^ii^	130.3 (6)
Ge1^ix^—In—Yb1^ii^	169.90 (3)	Ge2—Li1—Yb1^ii^	58.6 (4)
Ge2^x^—In—Yb1^ii^	60.68 (3)	Ge1^i^—Li1—Yb1^ii^	119.9 (9)
Ge2—In—Yb1^ii^	59.66 (3)	Ge1^ii^—Li1—Yb1^ii^	58.5 (3)
Li1^xi^—In—Yb1^ii^	129.7 (3)	In^x^—Li1—Yb1^ii^	65.5 (5)
Yb1^i^—In—Yb1^ii^	84.13 (4)	Li1^i^—Li1—Yb1^ii^	173.3 (13)
Ge1^viii^—In—Yb1^viii^	57.97 (4)	Li1^ii^—Li1—Yb1^ii^	95.2 (4)
Ge1^ix^—In—Yb1^viii^	118.63 (5)	Yb2^viii^—Li1—Yb1^ii^	67.4 (3)
Ge2^x^—In—Yb1^viii^	127.87 (3)	Yb2^ix^—Li1—Yb1^ii^	119.6 (8)
Ge2—In—Yb1^viii^	58.45 (3)	Yb2^v^—Li1—Yb1^ii^	117.3 (6)
Li1^xi^—In—Yb1^viii^	64.0 (4)	Ge1—Li1—Yb1^i^	130.3 (6)
Yb1^i^—In—Yb1^viii^	118.08 (3)	Ge2—Li1—Yb1^i^	58.6 (4)
Yb1^ii^—In—Yb1^viii^	67.29 (3)	Ge1^i^—Li1—Yb1^i^	58.5 (3)
Ge1^viii^—In—Yb1^ix^	118.63 (5)	Ge1^ii^—Li1—Yb1^i^	119.9 (9)
Ge1^ix^—In—Yb1^ix^	57.97 (4)	In^x^—Li1—Yb1^i^	65.5 (5)
Ge2^x^—In—Yb1^ix^	127.86 (3)	Li1^i^—Li1—Yb1^i^	95.2 (4)
Ge2—In—Yb1^ix^	58.45 (3)	Li1^ii^—Li1—Yb1^i^	173.3 (13)
Li1^xi^—In—Yb1^ix^	64.0 (4)	Yb2^viii^—Li1—Yb1^i^	119.6 (8)
Yb1^i^—In—Yb1^ix^	67.29 (3)	Yb2^ix^—Li1—Yb1^i^	67.4 (3)
Yb1^ii^—In—Yb1^ix^	118.08 (3)	Yb2^v^—Li1—Yb1^i^	117.3 (6)
Yb1^viii^—In—Yb1^ix^	79.49 (4)	Yb1^ii^—Li1—Yb1^i^	80.7 (6)

Symmetry codes: (i) −*x*, −*y*, −*z*+1; (ii) −*x*, −*y*+1, −*z*+1; (iii) −*x*+1/2, −*y*+1, *z*−1/2; (iv) −*x*+1/2, −*y*, *z*−1/2; (v) *x*−1/2, *y*, −*z*+1/2; (vi) *x*+1/2, *y*, −*z*+1/2; (vii) *x*, *y*, *z*−1; (viii) −*x*+1/2, −*y*+1, *z*+1/2; (ix) −*x*+1/2, −*y*, *z*+1/2; (x) *x*−1/2, *y*, −*z*+3/2; (xi) *x*+1/2, *y*, −*z*+3/2; (xii) *x*, *y*, *z*+1.
